# Bacteremia due to* Leuconostoc pseudomesenteroides* in a Patient with Acute Lymphoblastic Leukemia: Case Report and Review of the Literature

**DOI:** 10.1155/2016/7648628

**Published:** 2016-08-22

**Authors:** Kazuko Ino, Kazunori Nakase, Kei Suzuki, Akiko Nakamura, Atsushi Fujieda, Naoyuki Katayama

**Affiliations:** ^1^Department of Hematology and Oncology, Mie University Hospital, Tsu, Mie 514-8507, Japan; ^2^Cancer Center, Mie University Hospital, Tsu, Mie 514-8507, Japan; ^3^Emergency and Critical Care Center, Mie University Hospital, Tsu, Mie 514-8507, Japan; ^4^Central Clinical Laboratories, Mie University Hospital, Tsu, Mie 514-8507, Japan

## Abstract

*Leuconostoc* species are vancomycin-resistant Gram-positive cocci. Infections due to* Leuconostoc* species have been reported in various immunocompromised patients, but little is known about such infection in patients with hematologic malignancies. We report a case of* Leuconostoc *infection in a 44-year-old woman with acute lymphoblastic leukemia. The patient developed a high fever despite antimicrobial therapy with doripenem after induction chemotherapy. After an isolate from blood cultures was identified as* L. pseudomesenteroides*, we changed the antibiotics to piperacillin-tazobactam and gentamicin, after which the patient recovered from the infection. Physicians should be aware of* Leuconostoc* species as causative pathogen if they encounter Gram-positive cocci bacteremia resistant to standard antibiotics such as vancomycin and teicoplanin, especially in patients with hematologic malignancies.

## 1. Introduction

*Leuconostoc* species are Gram-positive cocci (GPC) that naturally reside in dairy products, vegetables, and legumes and occasionally in the human vagina and stool samples [1]. The prominent characteristic of* Leuconostoc* species is general resistance to glycopeptides, such as vancomycin (VCM) and teicoplanin (TEIC); this resistance is due to the absence of a target for these agents in the cell wall [2]. Since the first case of* Leuconostoc* infection in human was reported in 1985 [3], a variety of infections due to these bacteria have been reported in immunocompromised patients [1, 3]. However, little is known about this infection in patients with hematologic malignancies [4–6]. Here, we present a case of* Leuconostoc* bacteremia during chemotherapy in a patient with acute lymphoblastic leukemia (ALL) and review the literature describing its infection in hematologic patients.

## 2. Case Report

**Figure 1:**
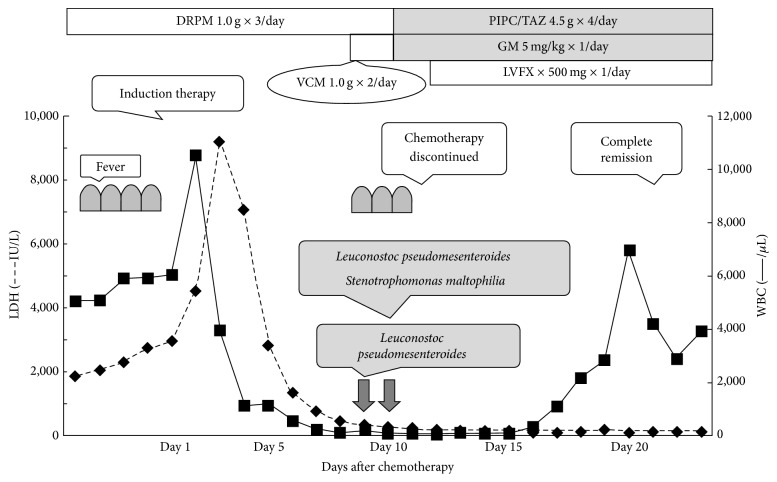
Clinical course of our case. Induction therapy consisted of cyclophosphamide 1,200 mg/m^2^ on day 1, doxorubicine 60 mg/m^2^ on days 1–3, vincristine 1.3 mg/m^2^ on days 1, 8, 15, and 22, L-asparaginase 3,000 U/m^2^ on days 9, 11, 13, 16, 18, and 20, and prednisolone 60 mg/m^2^ on days 1–21. DRPM, doripenem; VCM, vancomycin; PIPC/TAZ, piperacillin-tazobactam; GM, gentamicin; and LVFX, levofloxacin.

A 44-year-old woman was referred to our hospital because of a persistent fever and right-side back pain. On admission, her peripheral blood showed a hemoglobin level of 12.4 g/dL, platelet count of 2.7 × 10^4^/*μ*L, leukocyte count of 5,080/*μ*L with 17% abnormal lymphocytes, and an elevated lactate dehydrogenase level at 1,873 IU/L. The bone marrow was hypercellular, with 92% of myeloperoxidase-negative blast cells expressing CD10, CD19, CD20, CD34, and TdT. Cytogenetic analysis showed a complex karyotype without t(9; 22). The patient was diagnosed with precursor-B ALL and received induction chemotherapy comprising cyclophosphamide, doxorubicin, vincristine, L-asparaginase, and prednisolone (Figure 1). Since her admission (4 days before the initiation of chemotherapy), doripenem (DRPM) (1 g × 3/day) had been given because of a persistent fever suspected to be caused by a bacterial infection. Although the fever subsided within a week, DRPM continued even after that. Fluconazole (200 mg × 1/day, oral) was also used as antifungal prophylaxis. Five days after the initiation of chemotherapy (day 5), she became neutropenic (<500/*μ*L), and granulocyte colony stimulating factor (filgrastim, 300 *μ*g × 1/day, drip infusion) therapy was started. However, she developed a high fever, and two sets of blood cultures were positive for GPC on day 9. Consequently, we commenced VCM administration immediately. As her menstrual period overlapped the duration of the chemotherapy, her vaginal bleeding persisted and was accompanied by endometritis, which seemed to be the portal of entry for the etiological pathogen. Two days later, however, the isolate was confirmed as* L. pseudomesenteroides*, which is naturally resistant to VCM. Meanwhile, no organisms were identified from the culture of vaginal discharge. Considering the neutropenic status, we changed the antibiotics to piperacillin-tazobactam (4.5 g × 4/day) and gentamicin (5 mg/kg × 1/day) and removed the central venous catheter. Because the blood culture also identified* Stenotrophomonas maltophilia* on day 10, we also added levofloxacin (500 mg × 1/day). After receiving these three antibiotics for one week, the bacteria were cleared from the circulation, and the patient became afebrile. During this period, she achieved complete remission. The antibiotics were administered for 2 weeks.

## 3. Discussion

GPC bacteremia is increasingly recognized in neutropenic patients with acute leukemia during chemotherapy because of the frequent use of central venous catheter, oral antibacterial prophylaxis with fluoroquinolone, and chemotherapy-induced disruption of the intestinal mucosa [7]. Several guidelines for febrile neutropenia recommend early empirical treatment with VCM if GPC are detected in the blood culture [7, 8]. In our case, however, the VCM-resistant GPC,* L. pseudomesenteroides* was identified during the neutropenic period (Table 1). As TEIC susceptibility was not tested, the isolates' resistance to its agent was unclear. Nine cases of* Leuconostoc* infection have been previously reported in patients with hematologic malignancy (Table 2) [4–6, 9–12], including five cases of acute myeloid leukemia, three cases of non-Hodgkin's lymphoma, and one case of Hodgkin's lymphoma. Our case is the first ALL patient to be successfully treated. Notably, two (cases 1 and 7 in Table 2) of the nine hematologic cases died owing to inappropriate use of antibiotics [4, 5], though only a few deaths related to* Leuconostoc* species have been described in nonhematologic patients [5]. In case 1 with acute myeloid leukemia [4], a* Leuconostoc* species was isolated from blood cultures during the treatment of methicillin-resistant* Staphylococcus aureus* pneumonia with VCM. However, the organism was regarded as a contaminant of skin origin; thus VCM withdrawal was not justified because the patient was afebrile. As a result, the patient died from the progression of* Leuconostoc* infection. In case 7 with non-Hodgkin's lymphoma [5], the isolate was initially misdiagnosed as *α-Streptococcus*, and postmortem reevaluation of the blood culture confirmed that the GPC was a* Leuconostoc* species. Therefore, it is important to diagnose precisely and to administer the appropriate antibiotic promptly after confirmation of* Leuconostoc* species even if the patient is in an afebrile condition.

Recommended antibiotics for* Leuconostoc* infection include penicillin G, ampicillin, clindamycin, carbapenem, and/or aminoglycosides [13]. Consistent with our isolate showing a high minimum inhibitory concentration (MIC) to meropenem (Table 1), carbapenem-resistant isolates have also been described [14]. Recently, daptomycin has arisen as a promising agent for VCM-resistant GPC infection [9]. In the current patient, the susceptibility of the isolates to piperacillin-tazobactam and gentamicin could not be evaluated. Nevertheless, we selected these two agents under consideration of both anti-*Pseudomonas* and anti-*Leuconostoc* activities because the patient remained severely neutropenic. However, as the blood culture additionally yielded* S. maltophilia*, we also administered levofloxacin owing to a low MIC for this isolate. As levofloxacin had a low MIC for* L. pseudomesenteroides*, this agent might also exhibit an additive antibacterial effect for the* Leuconostoc* isolates.

In conclusion, VCM-resistant GPC should be considered as a causative pathogen in infections, even in patients with hematologic malignancy. Since* Leuconostoc* species are easily misidentified as* Streptococcus*,* Enterococcus*, or* Lactobacillus* [4, 5], it is particularly important to distinguish between these species. At present, proper management, including antibiotic therapy for this infection, has not yet been established. Accordingly, further accumulation of cases of* Leuconostoc* infection such as the present case is needed to better understand detailed clinical characteristics in patients with hematologic malignancies.

## Figures and Tables

**Table 1:** Minimal inhibitory concentration of isolates.

Antibiotics	*μ*g/mL
Penicillin G	1
Ampicillin	2
Cefotiam	>4
Cefotaxime	>4
Meropenem	>2
Erythromycin	<0.12
Clarithromycin	<0.12
Vancomycin	>1
Levofloxacin	1
Clindamycin	<0.12

**Table 2:** Clinical characteristics of hematologic patients that were reported to have *Leuconostoc* infection in the literature.

Case	Age/sex	Underlying disease	Condition	CVC (removal)	Identified bacteria from blood culture	Prior therapy	Treatment	Outcome
1	31/M	Acute myeloid leukemia	Pneumonia (postallogeneic BMT)	yes (yes)	*Leuconostoc* sp.	VCM	CAZ, CPFX	Died
2	18/F	Hodgkin's lymphoma	Septic shock, GVHD (postallogeneic BMT)	yes (yes)	*Leuconostoc mesenteroides*	VCM, IPM/CS → CPFX	DPT	Improved
3	35/M	Acute myeloid leukemia	Line-related bacteremia (postallogeneic BMT)	yes (yes)	*Leuconostoc mesenteroides*	VCM, CFPM, penicillin	DPT	Improved
4	25/F	Non-Hodgkin's lymphoma	Febrile neutropenia (after chemotherapy)	N/A	*Leuconostoc pseudomesenteroides Enterococcus faecium*	N/A	AMPC/CVA, GM, CPFX, ABPC	Improved
5	34/F	Acute myeloid leukemia	Febrile neutropenia, GVHD (postallogeneic PBSCT)	yes (N/A)	*Leuconostoc* sp.	TEIC	ABPC, GM	Improved
6	52/F	Acute myeloid leukemia	Right thigh cellulitis (after chemotherapy)	yes (N/A)	*Leuconostoc* sp. *Stenotrophomonas maltophilia*	VCM	IPM/CS, GM → ST, CLDM	Improved
7	73/M	Non-Hodgkin's lymphoma	Febrile neutropenia (after chemotherapy)	N/A	*Leuconostoc* sp.	PAPM/BP, GM	PAPM/BP, GM, VCM	Died
8	52/F	Acute myeloid leukemia (granulocytic sarcoma)	Febrile neutropenia (after chemotherapy)	yes (N/A)	*Leuconostoc lactis*	MEPM → CPFX, TEIC	LZD → TGC	Improved
9	64/M	Non-Hodgkin's lymphoma	Meningitis (after chemotherapy)	N/A	*Leuconostoc* sp.	CTRX → ABPC + GM	MEPM	Improved

BMT: bone marrow transplantation, GVHD: graft-versus-host disease, PBSCT: peripheral blood stem cell transplantation, CVC: central venous catheter, N/A: not applicable, sp.: species, VCM: vancomycin, IPM/CS: imipenem/cilastatin, CPFX: ciprofloxacin, CFPM: cefepime, TEIC: teicoplanin, PAPM/BM: panipenem/betamipron, GM: gentamycin, MEPM: meropenem, CTRX: ceftriaxone, ABPC: ampicillin, CAZ: ceftazidime, DPT: daptomycin, AMPC/CVA: amoxicillin/clavulanate, ST: sulfamethoxazole/trimethoprim, CLDM: clindamycin, LZD: linezolid, and TGC: tigecycline.
